# Cytokine gene polymorphism associations with congenital cytomegalovirus infection and sensorineural hearing loss

**DOI:** 10.1007/s10096-017-2996-6

**Published:** 2017-05-13

**Authors:** B. Kasztelewicz, J. Czech-Kowalska, B. Lipka, B. Milewska-Bobula, M. K. Borszewska-Kornacka, J. Romańska, K. Dzierżanowska-Fangrat

**Affiliations:** 1 Department of Clinical Microbiology and Immunology, The Children’s Memorial Health Institute, Dzieci Polskich 20, 04-730 Warsaw, Poland; 2 Department of Neonatology and Neonatal Intensive Care, The Children’s Memorial Health Institute, Warsaw, Poland; 3 Department of Infant Diseases, The Children’s Memorial Health Institute, Warsaw, Poland; 4 Department of Neonatology, Warsaw Medical University Hospital, Warsaw, Poland

## Abstract

Cytomegalovirus (CMV) is the most common viral agent of congenital infections and a leading nongenetic cause of sensorineural hearing loss (SNHL). The host immunologic factors that render a developing foetus prone to intrauterine CMV infection and development of hearing loss are unknown. The aim of this study was to assess the potential associations between the polymorphisms within cytokine and cytokine receptors genes, and the risk of congenital CMV infection, and the hearing outcome. A panel of 11 candidate single nucleotide polymorphisms (SNPs): TNF rs1799964, TNF rs1800629, TNFRSF1A rs4149570, IL1B rs16944, IL1B rs1143634, IL10 rs1800896, IL10RA rs4252279, IL12B rs3212227, CCL2 rs1024611, CCL2 rs13900, CCR5 rs333 was genotyped in 470 infants (72 with confirmed intrauterine CMV infection and 398 uninfected controls), and related to congenital CMV infection, and the outcome. In multivariate analysis, the IL1B rs16944 TT and TNF rs1799964 TC genotypes were significantly associated with intrauterine CMV infection (aOR = 2.32; 95% CI, 1.11–4.89; *p* = 0.032, and aOR = 2.17, 95% CI, 1.25–3.77; *p* = 0.007, respectively). Twenty-two out of 72 congenitally infected newborns had confirmed SNHL. Carriers of CT or TT genotype of CCL2 rs13900 had increased risk of hearing loss at birth and at 6 months of age (aOR = 3.59; *p* = 0.028 and aOR = 4.10; *p* = 0.039, respectively). This is the first study to report an association between SNPs in IL1B, TNF, and CCL2, and susceptibility to congenital CMV infection (IL1B and TNF) and SNHL (CCL2).

**Electronic supplementary material:**

The online version of this article (doi:10.1007/s10096-017-2996-6) contains supplementary material, which is available to authorized users.

## Introduction

Human cytomegalovirus (CMV) is a ubiquitous herpes virus. In the majority of healthy adults and children primary infection is asymptomatic, but CMV is an important cause of morbidity and mortality in immunocompromised individuals, and the most common cause of congenital infection worldwide, with a birth prevalence of 0.64% [1]. Maternal primary infection, reactivation of latent virus or re-infection with an antigenitically diverse strain during pregnancy, can all lead to in utero virus transmission to the developing foetus [2, 3]. Outcomes following congenital CMV infection are variable, ranging from asymptomatic infection and multiple organ involvement to stillbirth or death in the early neonatal period. Although only 10% of congenitally-infected neonates have obvious clinical signs at birth, approximately two-thirds of them have permanent neurologic sequelae [4, 5]. Moreover, even 15% of asymptomatic children have some type of long-term sequelae. Sensorineural hearing loss (SNHL) is the most common sequel following congenital CMV infection, affecting half of the symptomatic and 10–15% of the asymptomatic infants [6]. Early diagnosis and prompt intervention (i.e. treatment and rehabilitation) can reduce the long-term sequelae in congenitally infected newborns. However, this still remains challenging, as predictors of an adverse outcome, especially in neonates with an asymptomatic infection, have not been well defined.

It is arguable that both viral and host factors are implicated in the pathogenesis of congenital CMV infection. CMV has evolved mechanisms to evade and modulate immune response [7–9]. Evidence from recent studies suggests that CMV affects the cytokine profile within a CMV-infected placenta, at the maternal-foetal interference [10, 11]. CMV infection during pregnancy has been associated with a shift in cytokine expression toward a proinflammatory state [11]. This in turn may have important consequences for placental development and function, virus transmission, and foetus development [10]. Similarly, the host genetic background may influence the risk and the clinical manifestation of congenital CMV infection. An increasing body of evidence from studies performed mainly in immunocompromised subjects suggests that single nucleotide polymorphisms (SNPs) in genes implicated in immune response may have an impact on the course of CMV infection. A study by Loeffler et al. reported significant associations between SNPs in the IL10 and CCR5 genes, and CMV disease after allogeneic stem cell transplantation [12]. In the same study two SNPs within CCL2 gene encoding monocyte chemoattractant protein 1 were associated with CMV reactivation. Similarly, the 3’UTR polymorphism of the IL12B gene also has been linked with an increased risk of CMV reactivation after kidney transplant [13]. In another study, polymorphisms in the IL10RA gene encoding IL-10 receptor 1 were implicated in CMV retinitis among HIV-infected individuals [14]. Furthermore, a genetic variant in the promoter region of the TNF gene was associated with resistance to CMV infection in adult blood donors [15]. In addition, preliminary findings from studies analysing the role of cytokine polymorphisms in the setting of other herpes virus infections also suggested that genetic variants in IL1 complex, IL10, CCR5, TNF and TNFRSF1A genes could be associated with susceptibility to EBV infection or the pathogenesis of EBV-related diseases [16–21]. Whether polymorphisms in genes encoding cytokine or cytokine receptors contribute to the risk or the pathogenesis of congenital CMV remains unknown. Detailed knowledge of these polymorphisms in the context of congenital infection may have implications for clinical practice, by a possibility to predict the risk and/or the long-term outcome more accurately and, in turn, allowing appropriate neonatal counselling and the implementation of prompt intervention for newborns at high risk of an adverse outcome.

The aim of the present study was to evaluate the potential impact of the candidate SNPs in genes encoding cytokine and cytokine receptors on the risk of in utero CMV infection and its outcome.

## Materials and methods

### Study population

The study population consisted of 470 infants enrolled prospectively from March 2009 to July 2013 at the Children’s Memorial Health Institute in Warsaw, Poland. All subjects were of Caucasian background and were referred from other hospitals in the region. Seventy-two infants had congenital CMV infection confirmed by CMV DNA detection in urine samples collected during the first 2–3 weeks of life (case group). A healthy control group consisted of 398 CMV-uninfected children. All infants included in the study had whole blood sample available for a genotyping study. Children with probable postnatal infection (i.e. diagnosed after the third week of life), genetic disorders or congenital infections not related to CMV were excluded.

All infants with congenital CMV infection underwent complete physical examination, ophthalmologic and hearing evaluation, and cranial ultrasonography and/or computer tomography (CT). SNHL was defined as air conduction thresholds >25 dBHL on the auditory brainstem response (ABR) with normal bone conduction thresholds and normal middle ear function. Congenitally infected infants were classified as symptomatic on the basis of the presence of at least one of the following clinical findings at birth: petechiae, cholestatic jaundice (with direct bilirubin level > 2 mg/dL), hepatosplenomegaly, thrombocytopenia (<85,000 cells/mm^3^), intracranial calcification, microcephaly, seizures, chorioretinitis, or other abnormal CT findings (e.g. cerebral atrophy and/or malformations, periventricular cysts, ventriculomegaly). To evaluate the severity of congenital CMV disease an illness score was calculated for each child, one point being awarded for each sign and symptom.

### DNA isolation

Total genomic DNA was extracted from 200 μL of clinical specimen (whole blood, urine and cerebrospinal fluid) using QIAamp DNA Mini kit (Qiagen Inc., Hilden, Germany) according to the manufacturer’s instructions, with final elution of 100 μL.

### Detection of CMV DNA

The presence of CMV DNA was evaluated primarily in urine samples. In addition, children with congenital CMV infection (i.e. tested positive for CMV DNA in urine within the first 2–3 weeks of life) had CMV DNA tested in whole blood and some of them also in CSF, if clinically indicated. The presence of CMV DNA in clinical specimens was detected by qualitative real-time PCR using the SYBRGreen I format and primers set located in the lower matrix phosphoprotein (UL83) gene (Forward: 5′ TCTCGCACATCATGCTGGAT 3′ and Reverse: 5′ CGTTCATCAACAGGTTACCTGAGAT 3′). The PCR was performed on the 7500 Real-Time PCR System (Applied Biosystems, Inc., Foster City, CA, USA) in a total volume of 25 μL in the presence of 5 μL of DNA sample, 12.5 μL of SYBRGreen PCR MasterMix (Applied Biosystems) and 250 nM of each of the UL83 primers. The temperature profile was 95 °C for 10 min, 40 cycles at 95 °C for 15 s and 60 °C for 60 s. At the end of each run, a melting curve analysis was performed. The melting temperature range for CMV DNA positive samples was 81.5 ± 0.5 °C. The sensitivity of the assay was 0.99 copies/reaction.

### Determination of SNP genotypes

SNPs genotyping was performed using genomic DNA extracted from whole blood. Eleven SNPs in eight candidate genes were selected a priori on the basis of the following criteria: a minor allele frequency of >5% in the Pubmed SNP database (dbSNP) for Caucasian population (HapMap CEU or CAUC1), previous associations with infectious diseases or a plausible contribution to CMV diseases (i.e. SNPs within genes implicated in eliciting virus specific immune response and/or genes targeted by CMV in their immune evasion strategy). Briefly, TaqMan SNP Predesigned Genotyping Assays (Applied Biosystems, Inc., Foster City, CA, USA) were applied for: TNF rs1799964 (−1031 T/C), TNF rs1800629 (−308 G/A), TNFRSF1A rs4149570 (−201 C/A), CCL2 rs13900 (+1543 C/T), IL10 rs1800896 (−1082 A/G) and IL10RA rs4252279 (+5964 C/T) polymorphisms. The allelic discrimination was performed on the 7500 Real-time PCR System (Applied Biosystems) according to the manufacturer’s instructions. SNPs of IL1B rs16944 (−511C/T), IL1B rs1143634 (+3954 C/T), IL12B rs3212227 (3’UTR A/C), and CCL2 rs1024611 (−2518 A/G) were assessed by PCR and restriction fragment length polymorphism, as previously reported [13, 22–24]. The presence of 32-base pair deletions within the CCR5 gene (rs333, CCR5Δ32) were analysed by PCR [25]. A blinded duplicated analysis of 25 random study samples demonstrated 100% concordance.

### Statistical analysis

Quantitative data were expressed as mean ± standard deviation (SD) or the median and interquartile range (IQR) if non-normally distributed. Categorical variables were compared using the χ^2^ test or Fisher exact test. Continuous variables were compared using the Student t-test or Mann-Whitney U-test. All SNPs were analysed for Hardy-Weinberg equilibrium (HWE) by using the χ^2^ test (1 degree of freedom). The association between SNP genotype and congenital CMV infection or SNHL was analysed by co-dominant, dominant, recessive and over-dominant models. The genetic model that best fitted the data for each SNP was chosen based on the Akaike information criterion (AIC). The model with the lowest AIC value was the best model [26]. Odds ratios (ORs) with 95% confidence intervals (CIs) for each model were calculated using logistic regression. Linkage disequilibrium (LD) between multiple loci was measured using the standardized disequilibrium coefficient (D’) and the correlation coefficient (r^2^). The analysis haplotype frequencies was performed using the expectation-maximization algorithm. The association, HWE, LD and haplotype analyses were made by the SNPStats online tool [26]. Otherwise, statistical analyses were performed using Statistica software version 6.0 (StatSoft. Inc., Tulsa, USA). No correction was made for multiple testing. A two-tailed *p*-value of ≤0.05 was considered significant.

## Results

### Study population characteristics

The demographic and clinical characteristics of infants with congenital CMV infection (*n* = 72) and the uninfected control group (*n* = 398) are shown in Table 1. Infants with congenital CMV infection (as shown by detection of DNA in urine within the first 3 weeks of life) were more likely to be born to younger mothers. There were no differences between the two groups with respect to gender or gestational age (completed weeks).

**Table 1:** Characteristics of children with congenital CMV infection (*n* = 72) and healthy control group (*n* = 398)

Characteristics	Congenital CMV (*n* = 72)	Healthy control (*n* = 398)	*P*-value^a^
Male, *n* (%)	38 (52.7)	219 (55.0)	0.06
Gestational age, median (IQR), weeks	39 (37–40)	39 (38–40)	0.22
Prematurity (<37 weeks), (%)	13 (18.1)	82 (20.6)	0.62
Maternal age at delivery			
Mean (± SD), year	26.77 ± 4.63	29.89 ± 5.26	0.00001
Maternal CMV serostatus at delivery, (%)			NA
Naive	NA	75 (18.8)	
Immune	72 (100)	323 (81.2)	

*CMV* human cytomegalovirus, *IQR* interquartile range, *SD* standard deviation, *NA* not applicable

^a^χ^2^ test, Mann-Whitney U-test and Student’s t-test were performed when appropriate

At the time of the diagnosis, viral DNA was also detected in whole blood in 53 out of 72 infants with congenital CMV infection (73.6%) and in cerebrospinal fluid in 14 out of 56 tested (25%). At least one clinical finding suggestive of congenital CMV infection was found in 61 (84.7) infants (symptomatic CMV infection), whereas 11 (15.3%) infants were asymptomatic at birth. The illness scores in symptomatic infants ranged from 1 to 8 (median 3) (Table 2).

**Table 2:** Clinical and laboratory abnormalities at birth in infants with congenital CMV infection (*n* = 72)

Finding	Occurrences, *n* (%)
Cholestatic jaundice (direct bilirubin level > 2 mg/dL)	19 (26.4)
Petachial rash	17 (23.6)
Hepatosplenomegaly	26 (36.1)
Seizures	5 (6.9)
Thrombocytopenia (<85,000 cells/mm^3^)	30 (41.7)
Microcephaly	15 (20.8)
Intracranial calcifications	31 (43.1)
Other abnormal cranial findings^a^	45 (62.5)
Small for gestational age^b^	24 (33.3)
Number of findings (illness score)	
At least one finding (i.e. child was symptomatic)	61 (84.7)
1	15 (20.8)
2	14 (19.4)
≥3^c^	32 (44.4)

^a^Including: cerebral atrophy and/or malformations, periventricular cysts

^b^SGA, birth weight < 10th percentile for gestational age (children with SGA without other findings were considered as asymptomatic)

^c^The median illness score was 3; more specifically, an illness score of 3 was found in seven (9.7%) children, a score of 4 in eight (11.1%), score 5 in nine (12.5%), score 6 in four (5.6%), score 7 in three(4.2%) and score 8 in one (1.4%) child

**Fig. 1:**
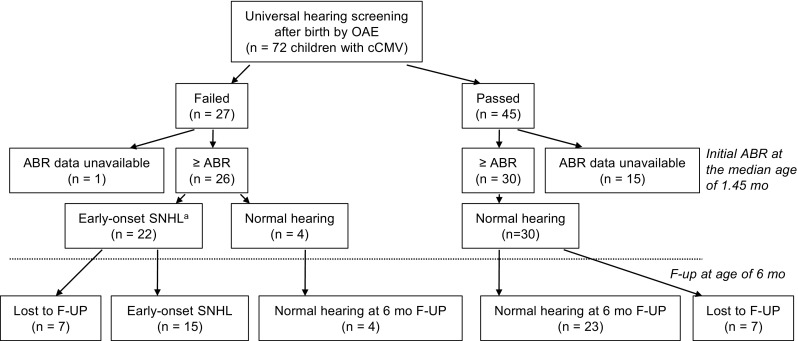
Overview of infants with congenital CMV infection according to hearing outcome. *OAE* otoacoustic emissions, *cCMV* congenital CMV infection, *F-UP* follow-up, *ABR* auditory brainstem response, *SNHL* sensorineural hearing loss ^a^ In all 22 children, ABR confirmed hearing loss in the same ear in which OAE testing failed, suggesting that hearing loss was present at birth; eight infants with unilateral SNHL (profound, *n* = 5; moderate, *n* = 2; and mild, *n* = 1) and 14 infants with bilateral SNHL (profound, *n* = 8; severe, *n* = 1; moderate, *n* = 4; mild, *n* = 1; all refer to the better ear). Hearing thresholds assessed with the ABR were defined as follows: a threshold of 0–25 dB for normal hearing, 26–40 dB for mild hearing loss, 41–60 dB for moderate hearing loss, 61–80 dB for severe hearing loss and >80 for profound hearing loss

The overview of hearing evaluation in infants with congenital CMV infection is shown in Fig. 1. All infants with congenital CMV infection had universal screening test by a transient otoacoustic emissions (OAE) test performed during the first month of life (median age of testing was 12 days, IQR: 2.5–32 days). Additionally, at least one ABR assessment (mean 3, range 1–10) was performed in 56 out of 72 infants with congenital CMV infection. The results of ABR were unavailable in 16 infants. The median age at initial ABR testing was 1.45 months (IQR: 1.1–3.5 months). Of the 72 infants with congenital CMV infection, 27 failed a universal screening test by OAE after birth. Twenty-two infants who failed a screening hearing test had confirmed early-onset SNHL by ABR (except one all these infants were symptomatic at birth), the remaining four had normal hearing (all symptomatic at birth) and one (asymptomatic at birth) had no ABR data available. In all 22 infants with SNHL, ABR confirmed hearing loss in the same ear in which OAE testing failed, suggesting that hearing loss was present at birth. Among 45 infants who passed a hearing screening test (36 symptomatic at birth), 30 infants had normal hearing thresholds in the ABR test (24 infants symptomatic at birth). Forty-two infants had follow-up data available at 6 months of age, i.e., in 15 infants, early-onset SNHL was diagnosed and 27 infants had normal hearing at 6 months of age.

### Results of SNP genotyping

Eleven candidate SNPs were genotyped in 470 children. All SNPs achieved over 99% successful genotyping. Genotype frequency distribution in children with congenital CMV and the healthy control group are listed in Supplementary Table S1. All SNPs were in HWE in a study population (*p* > 0.05).

### Associations of SNPs with in utero CMV infection

When genotype distributions in infants with congenital CMV infection were compared with uninfected controls, significant associations for IL1B rs16944 (IL1B -511 C/T) and TNF rs1799964 (−1031 C/T) polymorphisms were found (Table 3). The rare IL1B −511 TT genotype was overrepresented in infants with congenital CMV infection compared to controls (OR = 2.52; 95% CI, 1.25–5.08; *p* = 0.014). This association remained significant when adjusted for mother’s CMV status and age at delivery (OR = 2.32; 95% CI, 1.11–4.89; *p* = 0.032). Regarding TNF rs1799964 polymorphism, significantly higher frequency of heterozygous TC genotype was observed in infants with congenital CMV infection than in the control group (OR = 2.15; 95% CI, 1.27–3.63; *p* = 0.005 and OR = 2.17; 95% CI, 1.25–3.77; *p* = 0.007, for unadjusted and adjusted regression model, respectively). For other SNPs analysed, the genotype distributions were similar, both in the congenital CMV infection group and the controls (Supplementary Table S1).

**Table 3:** Associations of IL1B rs16944 (−511 C/T) and TNF rs1799964 (−1031 T/C) polymorphism with congenital CMV infection

Polymorphism SNP database ID number	Genotype	Children, n (%)		Unadjusted analysis		Adjusted analysis	
		Congenital CMV (*n* = 72)	Healthy control (*n* = 398)	OR (95% CI)	*P*-value	OR (95% CI)	*P*-value ^a^
IL1B rs16944	CC + CT	59 (81.9)	366 (92)	1	0.014	1	0.032
	TT	13 (18.1)	32 (8)	2.52 (1.25–5.08)		2.32 (1.11–4.89)	
TNF rs1799964	TT + CC	43 (59.7)	303 (76.1)	1	0.005	1	0.007
	TC	29 (40.3)	95 (23.9)	2.15 (1.27–3.63)		2.17 (1.25–3.77)	

^a^ *P*-value calculated for regression model adjusted for mother’s CMV status and age at delivery

### Association between SNP and hearing outcome in congenitally-infected infants

Taking into account the clinical outcome of congenital CMV infection, no significant association was found between severity of congenital CMV disease (i.e. illness score) and SNPs (Supplementary Table S2). However, when association analysis was performed according to hearing outcome at birth as well as at the age of 6 months, a significant relation between polymorphisms within CCL2 gene (rs13900) and SNHL was observed (Table 4). In particular, infants carrying CT or TT genotype of CCL2 rs13900 were statistically more likely to develop SNHL compared to infants with CC genotype (OR = 3.38; 95% CI, 1.06–10.74, *p* = 0.033 and OR = 4.0, 95% CI: 1.01–15.87, *p* = 0.04, for hearing status at birth and at the age of 6 months, respectively). These associations remained significant after adjusting for variable symptoms at birth (OR = 3.59; 95% CI, 1.10–11.73, *p* = 0.028 and OR = 4.10, 95% CI, 1.01–16.59, *p* = 0.039 for hearing status at birth and at the age of 6 months, respectively).

**Table 4:** Association of CCL2 rs13900 and rs1024611 polymorphisms with congenial CMV-related sensorineural hearing loss (SNHL) at birth and at the age of 6 months follow-up

Hearing status by ABR	CCL2 genotype ^a^	SNHL *n* (%)	Normal hearing *n* (%)	OR (95% CI)	*P* - value	OR (95% CI)	*P* - value ^b^
At birth (*n* = 56)							
rs13900	CC	6 (27.3)	19 (55.9)	1	0.033	1	0.028
	CT/TT	16 (72.7)	15 (44.1)	3.38 (1.06–10.74)		3.59 (1.10–11.73)	
rs1024611	AA	7 (31.8)	20 (58.8)	1	0.046		0.036
	AG/GG	15 (68.2)	14 (41.2)	3.06 (0.99–9.45)		3.33 (1.05–10.62)	
At the age of 6 months (*n* = 42)							
rs13900	CC	4 (26.7)	16 (59.3)	1	0.04	1	0.039
	CT/TT	11 (73.3)	11 (40.7)	4.0 (1.01–15.87)		4.10 (1.01–16.59)	
rs1024611	AA	5 (33.3)	17 (63)	1	0.064	1	0.057
	AG/GG	10 (66.7)	10 (37)	3.40 (0.90–12.83)		3.59 (0.93–13.90)	

^a^Strong linkage disequilibrium was observed between two SNP in the CCL2 gene rs13900 and rs1024611, (D’ = 1, r^2^ = 0.96)

^b^ *P*-value calculated for regression model adjusted for variable symptoms at birth

With regard to the second SNP within the CCL2 gene (rs1024611) analysed in the present study, an increased frequency of AG and GG genotypes was found in infants with SNHL when compared to those with normal hearing at birth (OR = 3.06; 95% CI, 0.99–9.45, *p* = 0.046 and OR = 3.33; 95% CI, 1.05–10.62, *p* = 0.036, for unadjusted and adjusted regression model, respectively). However, when association analysis was performed in a smaller group of infants with follow-up data available at the age of 6 month, the difference was not statistically significant (OR = 3.40; 95% CI, 0.90–12.83, *p* = 0.064 and OR = 3.59; 95% CI, 0.93–13.90, *p* = 0.057, for unadjusted and adjusted regression model, respectively). Given the close proximity of the two SNPs analysed within the CCL2 gene, linkage disequilibrium is highly probable. LD analysis confirmed a strong disequilibrium between rs1024611 and rs13900 SNPs in the CCL2 gene, with D’ = 1 and r^2^ = 0.96. Subsequently performed haplotype analysis revealed increased frequency of rs1024611-rs13900 G-T haplotype of CCL2 in infants with SNHL compared to the most frequent haplotype AC, but the difference was not statistically significant (OR = 2.33; 95% CI, 0.96–5.64, *p* = 0.067).

Genotype distribution of the remaining investigated SNPs did not show any significant difference between infants with congenital CMV infection and SNHL or normal hearing (Supplementary Table S3).

## Discussion

Genetic polymorphism in cytokine genes may lead to an altered immune response and, in turn, influence the risk or the outcome of an infectious disease [27]. While a number of investigators have reported relationships between genetic polymorphisms within cytokine or cytokine gene receptors and the CMV infection among immunosuppressed individuals, such as transplant recipients or AIDS patients [12–14], the role of such polymorphisms has not been studied in the setting of congenital CMV infection.

This is the first report on the relation between SNPs within cytokine and cytokine receptors and the risk of congenital CMV infection, and SNHL. Two novel observations arose from the present study. First, an association between SNPs rs16944 and rs1799964 located within the promoter region of IL1B (position 511) and TNF (position 1031) genes, respectively, and the susceptibility to in utero CMV infection were found. In particular, foetuses carrying a rare homozygous TT genotype of IL1B −511 are at over two-fold higher risk of congenital CMV infection compared to other IL1B −511 genotypes. Similarly, the heterozygous genotype of TNF rs1799964 was associated with an over two-fold increased risk of congenital CMV infection compared to other TNF −1031 genotypes. Taking into account the fact that the minor T allele of IL1B −511 renders high IL-1β producer phenotype with more severe and prolonged inflammatory response [28–30], and that polymorphism at −1031 of the TNF gene has been associated with enhanced expression of this cytokine [31], with increased frequency of these genotypes observed in congenitally infected children compared to healthy controls, suggests that a strong inflammatory response may make the foetus more prone to CMV infection. This is in line with previous studies from infected amniotic fluids and placentae showing that CMV infection leads to a pro-inflammatory (Th1) shift in cytokine profiles with a potential implication for the pathogenesis of foetal disease [10, 11]. However, due to pleiotropic properties of IL-1β and TNF-α, interactions between semi-allogenic placenta and the maternal immune system, the exact mechanism of the link between the genetic polymorphisms of IL1B and TNF genes, and the susceptibility to in utero CMV infection needs further examination in functional studies.

Second, a link between rs13900 polymorphisms within the CCL2 gene (encoding monocyte chemoattractant protein-1, MCP-1) and the hearing outcome of congenital CMV infection was observed. Carriers of minor homozygous and heterozygous genotypes of CCL2 rs13900 (at position +1543) were at significantly higher risk of developing SNHL as compared to common homozygotes. There was also association with CCL2 rs1024611 polymorphisms (at position −2518) and hearing outcome at birth. However this association failed to be significant when analyses were performed in a smaller group of infants with audiological data available at the age of 6 months. In accordance with the previous study, rs1024611 and rs13900 were in strong linkage disequilibrium [32], and subsequent haplotype analysis revealed a trend for increased frequency of rs1024611-rs13900 G-T haplotype of CCL2 in infants with SNHL. Recently, a robust functional study demonstrated that the haplotype containing the rs1024611G allele is associated with increased CCL2 expression [32]. Consistent with this, the polymorphisms in the CCL2 gene have been linked to increased MCP-1 levels in cerebrospinal fluid and monocyte infiltration of brain tissue in HIV-1 associated neurological disorders [33]. As the pathology of underlying SNHL is not fully understood, similarly the exact mechanism by which the polymorphisms in CCL2 might increase the risk of SNHL is currently unknown. Studies in animal models as well as findings in human temporal bones have consistently indicated that hearing loss following congenital CMV infection could have a significant inflammatory component [34, 35]. A recent study in a murine model of CMV-inducted hearing loss revealed that elevated expression of the subset of proinflammatory chemokines, which contribute to mononuclear cell infiltration into murine CMV infected cochlear cells rather than a virus mediated cytopathology, are responsible for hearing loss [34]. Further studies in a larger population of infants with SNHL as well as functional ones are required to confirm the link between the CCL2 polymorphisms and the risk of SNHL development. Until then, the mechanistic explanation of the potential link between SNPs in the cytokine genes and the outcome of congenital CMV infection presented here remains purely speculation.

If confirmed in larger prospective studies, these findings may have important implications. First, the interventions that modulate host inflammatory response could provide a promising therapeutic strategy for CMV infection. Second, the CCL2 genotype might be used as a diagnostic marker to identify individuals at risk of developing CMV-associated SNHL and perhaps to target them for early therapy and/or rehabilitation.

This study has some limitations. First, the rate of materno-foetal transmission may be influenced by gestational age at the time of maternal infection and viral load in the amniotic fluid, and these data were unavailable in this study. Second, as in the other studies of this type, it could not be excluded that the observed associations are due to a linkage with other, as yet unknown SNPs. Third, it should be noted that this data set was not adjusted for multiple testing, and so type 1 error cannot be completely ruled out. Finally, the analysis of association between SNPs in the CCL2 gene and hearing loss involved only infants with early-onset SNHL. These results require confirmation in large sized and longitudinal studies to be certain of the associations, as hearing loss in congenitally infected children may be progressive or have delayed onset.

In conclusion, the results of the present study suggest for the first time that polymorphisms in cytokine genes may influence the pathogenesis of congenital CMV infection. It has been shown that genetic polymorphisms in IL1B and TNF may modulate the susceptibility to intrauterine CMV infection, whereas CCL2 polymorphisms may influence the hearing outcome in children with congenital CMV infection. Further, larger studies and functional assessment of the SNPs are required to confirm their pathogenic role.

## Electronic supplementary material

ESM 1 (PDF 300 kb)
