# Supplementary material for: Cytokine gene polymorphism associations with congenital cytomegalovirus infection and sensorineural hearing loss
Source: Eur J Clin Microbiol Infect Dis. 2017 May 13;36(10):1811–8. doi: 10.1007/s10096-017-2996-6 (PMC5602083; doi:10.1007/s10096-017-2996-6)
Supplement: Supplementary file 1 — (PDF 300 kb) [file 10096_2017_2996_MOESM1_ESM.pdf]

Cytokine gene polymorphisms associations with congenital cytomegalovirus infection and sensorineural hearing loss

European Journal of Clinical Microbiology &Infectious Diseases

Beata Kasztelewicz,[1] PhD; Justyna Czech-Kowalska,[2] PhD; Bożena Lipka,[3] PhD; Bogumiła Milewska-Bobula,[3] Prof.; Maria K. Borszewska-Kornacka,[4] Prof.; Justyna Romańska,[4] PhD; Katarzyna Dzierżanowska-Fangrat,[1] PhD

[1] Department of Clinical Microbiology and Immunology, The Children's Memorial Health Institute, Warsaw, Poland.

[2] Department of Neonatology and Neonatal Intensive Care, The Children's Memorial Health Institute, Warsaw, Poland.

[3] Department of Infant Diseases, The Children's Memorial Health Institute, Warsaw, Poland.

[4] Department of Neonatology, Warsaw Medical University Hospital, Warsaw, Poland.

Corresponding author: Beata Kasztelewicz, e-mail address: b.kasztelewicz@czd.pl

Table S1 Association analysis of individual SNPs with congenital CMV infection

Gene (SNP database ID number)	Genetic model	Genotype	Genotype frequencies; n (%) Congenital CMV (n = 72)	Healthy controls (n = 398)	OR (95% CI)	P-value[a]	AIC
**IL1B rs16944** (-511 T/C)	Co-D	CC	31 (43.1)	183 (46.0)	1	**0.046**	402.4
		CT	28 (38.9)	183 (46.0)	0.90 (0.52 - 1.57)		
		TT	13 (18.1)	32 (8.0)	2.40 (1.13 - 5.07)		
	D	CC	31 (43.1)	183 (46.0)	1	0.65	406.3
		CT/TT	41 (56.9)	215 (54.0)	1.13 (0.68 - 1.87)		
	R	CC/CT	59 (81.9)	366 (92.0)	1	**0.014**	400.5
		TT	13 (18.1)	32 (8.0)	2.25 (1.25 - 5.08)		
	O-D	CC/TT	44 (61.1)	215 (54.0)	1	0.26	405.3
		CT	28 (38.9)	183 (46.0)	0.75 (0.45 - 1.25)		
**IL1B rs1143634** (+3954 T/C)	Co-D	CC	43 (59.7)	277 (57.0)	1	0.91	408.3
		CT	24 (33.3)	141 (35.4)	0.90 (0.52 - 1.54)		
		TT	5 (6.9)	30 (7.5)	0.88 (0.32 - 2.39)		
	D	CC	43 (59.7)	227 (57.0)	1	0.67	406.3
		CT/TT	29 (40.3)	171 (43.0)	0.90 (0.54 - 1.49)		
	R	CC/CT	67 (93.1)	368 (92.5)	1	0.86	406.5
		TT	5 (6.9)	30 (7.5)	0.92 (0.34 - 2.44)		
	O-D	CC/TT	48 (66.7)	257 (64.6)	1	0.73	406.4
		CT	24 (33.3)	141 (35.4)	0.91 (0.54 - 1.55)		
**IL12B rs3212227** (3'UTR C/A)	Co-D	AA	49 (68.1)	255 (64.1)	1	0.53	407.2
		AC	22 (30.6)	129 (32.4)	0.89 (0.51 - 1.53)		
		CC	1 (1.4)	14 (3.5)	0.37 (0.05 - 2.89)		
	D	AA	49 (68.1)	255 (64.1)	1	0.51	406.1
		AC/CC	23 (31.9)	143 (35.9)	0.84 (0.49 - 1.43)		
	R	AA/AC	71 (98.6)	384 (96.5)	1	0.3	405.4
		CC	1 (1.4)	14 (3.5)	0.39 (0.05 - 2.98		
	O-D	AA/CC	50 (69.4)	269 (67.6)	1	0.76	406.4

		AC	22 (30.6)	129 (32.4)	0.92 (0.53 - 1.58)		
**CCL2 rs13900** [b] (+1543 T/C)	Co-D	CC<br>CT<br>TT	35 (48.6)<br>30 (41.7)<br>7 (9.7)	208 (52.4)<br>151 (38.0)<br>38 (9.6)	1<br>1.18 (0.69 - 2.01)<br>1.09 (0.45 - 2.64)	0.83	407.8
	D	CC<br>CT/TT	35 (48.6)<br>37 (51.4)	208 (52.4)<br>189 (47.6)	1<br>1.16 (0.70 - 1.92)	0.55	405.8
	R	CC/CT<br>TT	65 (90.3)<br>7 (9.7)	359 (90.4)<br>38 (9.6)	1<br>1.02 (0.44 - 2.38)	0.97	406.2
	O-D	CC/TT<br>CT	42 (58.3)<br>30 (41.7)	246 (62.0)<br>151 (38.0)	1<br>1.16 (0.70 - 1.94)	0.56	405.8
**CCL2 rs1024611** [c] (-2518 G/A)	Co-D	AA<br>AG<br>GG	36 (50)<br>29 (40.3)<br>7 (9.7)	219 (55.4)<br>142 (36.0)<br>34 (8.6)	1<br>1.24 (0.73 - 2.12)<br>1.25 (0.52 - 3.04)	0.7	406.8
	D	AA<br>AG/GG	36 (50)<br>36 (50)	219 (55.4)<br>176 (44.6)	1<br>1.24 (0.75 - 2.06)	0.39	404.8
	R	AA/AG<br>GG	65 (90.3)<br>7 (9.7)	361 (91.4)<br>34 (8.6)	1<br>1.14 (0.49 - 2.69)	0.76	405.4
	O-D	AA/GG<br>AG	43 (59.7)<br>29 (40.3)	253 (64.0)<br>142 (36.0)	1<br>1.20 (0.72 - 2.01)	0.49	405.0
**CCR5 rs333** (CCR5Δ32)	Co-D	wt wt<br>wtΔ32<br>Δ32Δ32	58 (80.6)<br>14 (19.4)<br>0 (0)	334 (83.9)<br>63 (15.8)<br>1 (0.2)	1<br>1.28 (0.67 - 2.43)<br>0.00 (0.00 - NA)	0.64	407.6
	D	wtwt<br>wtΔ32/Δ32Δ32	58 (80.6)<br>14 (19.4)	334 (83.9)<br>64 (16.1)	1<br>1.26 (0.66 - 2.39)	0.49	406.0
	R	wtwt/wtΔ32<br>Δ32Δ32	72 (100)<br>0 (0)	397 (99.8)<br>1 (0.2)	1<br>0.00 (0.00 - NA)	0.56	406.2
	O-D	wtwt/Δ32Δ32<br>wtΔ32	58 (80.6)<br>14 (19.4)	335 (84.2)<br>63 (15.8)	1<br>1.28 (0.67 - 2.44)	0.45	406.0
**TNF rs1799964** (-1031 C/T)	Co-D	TT<br>TC	42 (58.3)<br>29 (40.3)	283 (71.1)<br>95 (23.9)	1<br>2.06 (1.21 - 3.48)	**0.0092**	399.1

		CC	1 (1.4)	20 (5.0)	0.34 (0.04 - 2.58)		
	D	TT	42 (58.3)	283 (71.1)	1	**0.034**	402.0
		TC/CC	30 (41.7)	115 (28.9)	1.76 (1.05 - 2.95)		
	R	TT/TC	71 (98.6)	378 (95.0)	1	0.12	404.1
		CC	1 (1.4)	20 (5.0)	0.27 (0.04 - 2.02)		
	O-D	TT/CC	43 (59.7)	303 (76.1)	1	**0.005**	398.6
		TC	29 (40.3)	95 (23.9)	2.15 (1.27 - 3.63)		
**TNF rs1800629**	Co-D	GG	52 (72.2)	283(71.1)	1	0.43	406.8
(-308 A/G)		GA	17 (23.6)	108 (27.1)	0.86 (0.47 - 1.55)		
		AA	3 (4.2)	7 (1.8)	2.33 (0.58 - 9.31)		
	D	GG	52 (72.2)	283 (71.1)	1	0.85	406.5
		GA/AA	20 (27.8)	115 (28.9)	0.95 (0.54 - 1.66)		
	R	GG/GA	69 (95.8)	391 (98.2)	1	0.24	405.1
		AA	3 (4.2)	7 (1.8)	2.43 (0.61 - 9.62)		
	O-D	GG/AA	55 (76.4)	290 (72.9)	1	0.53	406.1
		GA	17 (23.6)	108 (27.1)	0.83 (0.46 - 1.49)		
**TNFRSF1A rs4149570** (-201 A/C)	Co-D	CC	29 (40.3)	173 (43.5)	1	0.55	407.3
		CA	35 (48.6)	168 (42.2)	1.24 (0.73 - 2.12)		
		AA	8 (11.1)	57 (14.3)	0.84 (0.36 - 1.94)		
	D	CC	29 (40.3)	173 (43.5)	1	0.61	406.3
		CA/CC	43 (59.7)	225 (56.5)	1.14 (0.68 - 1.90)		
	R	CC/CA	64 (88.9)	341 (85.7)	1	0.46	406.0
		AA	8 (11.1)	57 (14.3)	0.75 (0.34 - 1.64)		
	O-D	CC/AA	37 (51.4)	230 (57.8)	1	0.31	405.5
		CA	35 (48.6)	168 (42.2)	1.30 (0.78 - 2.14)		
**IL10 rs1800896**	Co-D	TT	23 (31.9)	127 (31.9)	1	0.11	404.2
(-1082 G/A)		CT	42 (58.3)	196 (49.2)	1.18 (0.68 - 2.06)		
		CC	7 (9.7)	75 (18.8)	0.52 (0.21 - 1.26)		
	D	TT	23 (31.9)	127 (31.9)	1	1	406.5
		CT/CC	49 (68.1)	271 (68.1)	1.00 (0.58 - 1.71)		

	R	TT/CT<br>CC	65 (90.3)<br>7 (9.7)	323 (81.2)<br>75 (18.8)	1<br>0.46 (0.20 - 1.05)	0.046	402.5
	O-D	TT/CC<br>CT	30 (41.7)<br>42 (58.3)	202 (50.8)<br>196 (49.2)	1<br>1.44 (0.87 - 2.40)	0.15	404.5
**IL10RA rs4252279**<br>(+5964 T/C)	Co-D	CC<br>CT<br>TT	54 (75)<br>16 (22.2)<br>2 (2.8)	307 (77.1)<br>90 (22.6)<br>1 (0.2)	1<br>1.01 (0.55 - 1.85)<br>11.37 (1.01 - 127.60)	0.13	404.5
	D	CC<br>CT/TT	54 (75)<br>18 (25)	307 (77.1)<br>91 (22.9)	1<br>1.12 (0.63 - 2.01)	0.69	406.4
	R	CC/CT<br>TT	70 (97.2)<br>2 (2.8)	397 (99.8)<br>1 (0.2)	1<br>11.34 (1.01 - 126.79)	0.044	402.5
	O-D	CC/TT<br>CT	56 (77.8)<br>16 (22.2)	308 (77.4)<br>90 (22.6)	1<br>0.98 (0.53 - 1.79)	0.97	406.5

Abbreviations: AIC, Akaike information criterion; Co-D, Co-dominat; D, Dominant; R, Recessive; O-D, Over-dominant; IL1B. interleukin 1 beta; IL12B. interleukin 12B; 3'UTR. 3'untranslated region; CCL2. chemokine (C-C motif) ligand 2; CCR5. chemokine (C-C motif) receptor 5 gene; wt - wild type; Δ32. 32-base pair deletion; NA. not applicable; TNF. tumour necrosis factor; TNFRSF1A. tumour necrosis factor receptor superfamily member 1A. IL10. interleukin 10; IL10RA. interleukin 10 receptor alpha.

[a] Significance levels were assessed using logistic regression model

[b] Percentage of genotyped samples was 469/470 (99.8%)

[c] Percentage of genotyped samples was 467/470 (99.4%)

Table S2 Association analysis of individual SNPs with severity of congenital CMV disease (i.e. illness score)

**Gene**<br>**(SNP database ID number)**	**Genotype**	**N**	**Mean illness score (SE)**	**Difference (95% CI)**	**P-value** [a]
**IL1B rs16944**	CC	31	2.58 (0.35)	0	0.15
(-511 T/C)	CT	28	3.21 (0.44)	0.63 (-0.44 - 1.70)	
	TT	13	1.85 (3.5)	-0.73 (-2.09 - 0.62	
**IL1B rs1143634**	CC	43	2.44 (0.29)	0	0.41
(+3954 T/C)	CT	24	3.17 (0.48)	0.72 (-0.34 - 1.79)	
	TT	5	2.6 (1.21)	0.16 (-1.81 - 2.21)	
**IL12B rs3212227**	AA	49	2.69 (0.31)	0	0.12
(3'UTR C/A)	AC	22	2.5 (0.39)	-0.19 (-1.24 - 0.85)	
	CC	1	7 (0)	4.31 (0.18 - 8.43)	
**CCL2 rs13900**	CC	35	2.29 (0.34)	0	0.27
(+1543 T/C)	CT	30	3.13 (0.37)	0.85 (-0.18 - 1.88)	
	TT	7	2.86 (1.12)	0.57 (-1.14 - 2.28)	
**CCL2 rs1024611**	AA	36	2.36 (0.34)	0	0.41
(-2518 G/A)	AG	29	3.07 (0.37)	0.71 (-0.33 - 1.75)	
	GG	7	2.86 (1.12)	0.50 (-1.22 - 2.21)	
**CCR5 rs333**	wt wt	58	2.64 (0.28)	0	0.65
(CCR5Δ32)	wtΔ32	14	2.93 (0.55)	0.29 (-0.95 - 1.54)	
**TNF rs1799964**	TT	42	2.69 (0.3)	0	0.44
(-1031 C/T)	TC	29	2.79 (0.44)	0.10 (-0.90 - 1.11)	
	CC	1	0 (0)	-2.69 (-6.91 - 1.52)	
**TNF rs1800629**	GG	52	2.52 (0.29)	0	0.14
(-308 A/G)	GA	17	2.82 (0.51)	0.30 (-0.84 - 1.45)	
	AA	3	5 (1)	2.48 (0.05 - 4.91)	
**TNFRSF1A rs4149570**	CC	29	3 (0.42)	0	0.61
(-201 A/C)	CA	35	2.49 (0.32)	-0.51 (-1.57 - 0.54)	
	AA	8	2.5 (0.94)	-0.50 (-2.17 - 1.17)	
**IL10 rs1800896**	TT	23	3.3 (0.44)	0	0.25

(-1082 G/A)	CT	42	2.43 (0.33)	-0.88 (-1.95 - 0.20)	
	CC	7	2.29 (0.75)	-1.02 (-2.80 - 0.76)	
**IL10RA rs4252279**	CC	54	2.74 (0.29)	0	0.19
(+5964 T/C)	CT	16	2.88 (0.52)	0.13 (-1.04 - 1.31)	
	TT	2	0 (0)	-2.74 (-5.70 - 0.22)	

[a] P-value calculated for linear regression.

Table S3 Association analysis of individual SNPs with congenital CMV-related sensorineural hearing loss (SNHL) at birth

Gene (SNP database ID number)	Genetic model	Genotype	Genotype frequencies; n (%) SNHL (n = 22)	Normal hearing (n = 34)	OR (95% CI)	P-value[a]	AIC
**IL1B rs16944** (-511 T/C)	Co-D	CC	9 (40.9)	14 (41.2)	1	0.94	80.9
		CT	9 (40.9)	15 (44.1)	0.93 (0.29 - 3.03)		
		TT	4 (18.2)	5 (14.7)	1.24 (0.26 - 5.92)		
	D	CC	9 (40.9)	14 (41.2)	1	0.98	79
		CT/TT	13 (59.1)	20 (58.8)	1.01 (0.34 - 3.01)		
	R	CC/CT	18 (81.8)	29 (85.3)	1	0.73	78.9
		TT	4 (18.2)	5 (14.7)	1.29 (0.31 - 5.44)		
	O-D	CC/TT	13 (59.1)	19 (55.9)	1	0.81	79
		CT	9 (40.9)	15 (44.1)	0.88 (0.30 - 2.60)		
**IL1B rs1143634** (+3954 T/C)	Co-D	CC	12 (54.5)	23 (67.7)	1	0.15	77.3
		CT	10 (45.5)	9 (26.5)	2.13 (0.68 - 6.65)		
		TT	0 (0)	2 (5.9)	0 (0.00 - NA)		
	D	CC	12 (54.5)	23 (67.7)	1	0.32	78.1
		CT/TT	10 (45.5)	11 (32.4)	1.74 (0.58 - 5.26)		
	R	CC/CT	22 (100)	32 (94.1)	1	0.15	77
		TT	0 (0)	2 (5.9)	0 (0.00 - NA)		
	O-D	CC/TT	12 (54.5)	25 (73.5)	1	0.14	76.9
		CT	10 (45.5)	9 (26.5)	2.31 (0.75 - 7.19)		
**IL12B rs3212227** (3'UTR C/A)	Co-D	AA	14 (63.6)	23 (67.7)	1	0.54	79.8
		AC	8 (36.4)	10 (29.4)	1.31 (0.42 - 4.12)		
		CC	0 (0)	1 (2.9)	0 (0.00 - NA)		
	D	AA	14 (63.6)	23 (67.7)	1	0.76	78.9
		AC/CC	8 (36.4)	11 (32.4)	1.19 (0.39 - 3.69)		
	R	AA/AC	22 (100)	33 (97.1)	1	0.32	78
		CC	0 (0)	1 (2.9)	0 (0.00 - NA)		

	O-D	AA/CC<br>AC	14 (63.6)<br>8 (36.4)	24 (70.6)<br>10 (29.4)	1<br>1.37 (0.44 - 4.29)	0.59	78.7
**CCL2 rs13900**<br>(+1543 T/C)	Co-D	CC<br>CT<br>TT	6 (27.3)<br>13 (59.1)<br>3 (13.6)	19 (55.9)<br>12 (35.3)<br>3 (8.8)	1<br>3.43 (1.03 - 11.48)<br>3.17 (0.50 - 20.04)	0.1	76.5
	D	CC<br>CT/TT	6 (27.3)<br>16 (72.7)	19 (55.9)<br>15 (44.1)	1<br>3.38 (1.06 - 10.74)	**0.033**	74.5
	R	CC/CT<br>TT	19 (86.4)<br>3 (13.6)	31 (91.2)<br>3 (8.8)	1<br>1.63 (0.30 - 8.92)	0.57	78.7
	O-D	CC/TT<br>CT	9 (40.9)<br>13 (59.1)	22 (64.7)<br>12 (35.3)	1<br>2.65 (0.88 - 7.98)	0.08	76
**CCL2 rs1024611**<br>(-2518 G/A)	Co-D	AA<br>AG<br>GG	7 (31.8)<br>12 (54.5)<br>3 (13.6)	20 (58.8)<br>11 (32.4)<br>3 (8.8)	1<br>3.12 (0.95 - 10.22)<br>2.86 (0.46 - 17.58)	0.14	77.1
	D	AA<br>AG/GG	7 (31.8)<br>15 (68.2)	20 (58.8)<br>14 (41.2)	1<br>3.06 (0.99 - 9.45)	0.046	75.1
	R	AA/AG<br>GG	19 (86.4)<br>3 (13.6)	31 (91.2)<br>3 (8.8)	1<br>1.63 (0.30 - 8.92)	0.57	78.7
	O-D	AA/GG<br>AG	23 (67.7)<br>11 (32.4)	10 (45.5)<br>12 (54.5)	1<br>2.51 (0.83 - 7.57)	0.099	76.3
**CCR5 rs333**<br>(CCR5Δ32)	NA	wt wt<br>wtΔ32	17 (77.3)<br>5 (22.7)	28 (82.3)<br>6 (17.6)	1<br>1.37 (0.36 - 5.20)	0.64	78.8
**TNF rs1799964**<br>(-1031 C/T)	Co-D	TT<br>TC<br>CC	12 (54.5)<br>10 (45.5)<br>0 (0)	19 (55.9)<br>14 (41.2)<br>1 (2.9)	1<br>1.13 (0.38 - 3.35)<br>0 (0.00 - NA)	0.59	80
	D	TT<br>TC/CC	12 (54.4)<br>10 (45.5)	19 (55.9)<br>15 (44.1)	1<br>1.06 (0.36 - 3.10)	0.92	79
	R	TT/TC<br>CC	22 (100)<br>0 (0)	33 (97.1)<br>1 (2.9)	1<br>0 (0.00 - NA)	0.32	78
	O-D	TT/CC	12 (54.4)	20 (58.8)	1	0.75	78.9

		TC	10 (45.5)	14 (41.2)	1.19 (0.40 - 3.51)		
**TNF rs1800629** (-308 A/G)	Co-D	GG<br>GA<br>AA	16 (72.7)<br>5 (22.7)<br>1 (4.5)	26 (76.5)<br>7 (20.6)<br>1 (2.9)	1<br>1.16 (0.31 - 4.28)<br>1.62 (0.09 - 27.84)	0.93	80.9
	D	GG<br>GA/AA	16 (72.7)<br>6 (27.3)	26 (76.5)<br>8 (23.5)	1<br>1.22 (0.36 - 4.16)	0.75	78.9
	R	GG/GA<br>AA	21 (95.5)<br>1 (4.5)	33 (97.1)<br>1 (2.9)	1<br>1.57 (0.09 - 26.50)	0.75	78.9
	O-D	GG/AA<br>GA	17 (77.3)<br>5 (22.7)	27 (79.4)<br>7 (20.6)	1<br>1.13 (0.31 - 4.16)	0.85	79
**TNFRSF1A rs4149570** (-201 A/C)	Co-D	CC<br>CA<br>AA	11 (50)<br>9 (40.9)<br>2 (9.1)	12 (35.3)<br>17 (50)<br>5 (14.7)	1<br>0.58 (0.18 - 1.82)<br>0.44 (0.07 - 2.73)	0.53	79.8
	D	CC<br>CA/CC	11 (50)<br>11 (50)	12 (35.3)<br>22 (64.7)	1<br>0.55 (0.18 - 1.63)	0.28	77.9
	R	CC/CA<br>AA	20 (90.9)<br>2 (9.1)	29 (85.3)<br>5 (14.7)	1<br>0.58 (0.10 - 3.29)	0.53	78.6
	O-D	CC/AA<br>CA	13 (59.1)<br>9 (40.9)	17 (50)<br>17 (50)	1<br>0.69 (0.23 - 2.05)	0.5	78.6
**IL10 rs1800896** (-1082 G/A)	Co-D	TT<br>CT<br>CC	9 (40.9)<br>11 (50)<br>2 (9.1)	13 (38.2)<br>20 (58.8)<br>1 (2.9)	1<br>0.79 (0.26 - 2.44)<br>2.89 (0.23 - 36.87)	0.57	79.9
	D	TT<br>CT/CC	9 (40.9)<br>13 (59.1)	13 (38.2)<br>21 (61.8)	1<br>0.89 (0.30 - 2.68)	0.84	79
	R	TT/CT<br>CC	20 (90.9)<br>2 (9.1)	33 (97.1)<br>1 (2.9)	1<br>3.30 (0.28 - 38.78)	0.32	78.1
	O-D	TT/CC<br>CT	11 (50)<br>11 (50)	14 (41.2)<br>20 (58.8)	1<br>0.70 (0.24 - 2.06)	0.52	78.6
**IL10RA rs4252279** (+5964 T/C)	Co-D	CC<br>CT	17 (77.3)<br>5 (22.7)	23 (67.7)<br>9 (26.5)	1<br>0.75 (0.21 - 2.65)	0.33	78.8

		TT	0 (0)	2 (5.9)	0 (0.00 - NA)		
	D	CC	17 (77.3)	23 (67.7)	1	0.43	78.4
		CT/TT	5 (22.7)	11 (32.4)	0.61 (0.18 - 2.10)		
	R	CC/CT	22 (100)	32 (94.1)	1	0.15	77
		TT	0 (0)	2 (5.9)	0 (0.00 - NA)		
	O-D	CC/TT	17 (77.3)	25 (73.5)	1	0.75	78.9
		CT	5 (22.7)	9 (26.5)	0.82 (0.23 - 2.87)		

[a] Significance levels were assessed using logistic regression model
